# Multi-Night Home Assessment of Total Sleep Time Misperception in Obstructive Sleep Apnea with and Without Insomnia Symptoms

**DOI:** 10.3390/clockssleep6040050

**Published:** 2024-12-05

**Authors:** Jasmin Kuhn, Laura R. B. Schiphorst, Bernice M. Wulterkens, Jerryll Asin, Nanny Duis, Sebastiaan Overeem, Merel M. van Gilst, Pedro Fonseca

**Affiliations:** 1 Department of Electrical Engineering, Eindhoven University of Technology, 5612AP Eindhoven, The Netherlands; j.kuhn@tue.nl (J.K.); l.r.b.schiphorst@tue.nl (L.R.B.S.); b.m.wulterkens@tue.nl (B.M.W.); m.m.v.gilst@tue.nl (M.M.v.G.); pedro.fonseca@philips.com (P.F.); 2 Philips Sleep and Respiratory Care, 5656AE Eindhoven, The Netherlands; 3 Center for Sleep Medicine, Amphia Hospital, 4818CK Breda, The Netherlands; jasin@amphia.nl (J.A.);; 4 Sleep Medicine Center Kempenhaeghe, 5591VE Heeze, The Netherlands

**Keywords:** obstructive sleep apnea, sleep disordered breathing, insomnia, sleep perception, sleep-wake cognition, photoplethysmography, repeated measurements

## Abstract

Total sleep time (TST) misperception has been reported in obstructive sleep apnea (OSA). However, previous findings on predictors were inconsistent and predominantly relied on single-night polysomnography, which may alter patients’ sleep perception. We leveraged advances in wearable sleep staging to investigate predictors of TST misperception in OSA over multiple nights in the home environment. The study included 141 patients with OSA, 75 without insomnia symptoms (OSA group), and 66 with insomnia symptoms (OSA-I group). Objective TST was measured using a previously validated wrist-worn photoplethysmography and accelerometry device. Self-reported TST was assessed using a digital sleep diary. TST misperception was quantified with the misperception index (MI), calculated as (objective − self-reported TST)/objective TST. MI values differed significantly between the OSA (median = −0.02, IQR = [−0.06, 0.02]) and the OSA-I group (0.05, [−0.02, 0.13], *p* < 0.001). Multilevel modeling revealed that the presence of insomnia symptoms (β = 0.070, *p* < 0.001) and lower daily reported sleep quality (β = −0.229, *p* < 0.001) were predictive of higher MI (TST underestimation), while a higher apnea–hypopnea index (AHI) was predictive of lower MI (TST overestimation; β = −0.001, *p* = 0.006). Thus, insomnia symptoms and AHI are associated with TST misperception in OSA patients, but in opposite directions. This association extends over multiple nights in the home environment.

## 1. Introduction

A discrepancy between the subjective experience and objective recording of sleep duration is often described as total sleep time (TST) misperception. It is regularly encountered in clinical practice and many sleep-disordered patients tend to underestimate their sleep time [1,2,3]. This is clinically relevant since patients who believe that they are getting less sleep than they actually are may become anxious about their perceived lack of sleep. This can hinder their propensity to sleep, leading to a vicious cycle further impairing sleep [4,5]. Consequently, sleep misperception is associated with a lower quality of life [6], impaired daytime functioning [7], and greater functional disability [8]. Historically, TST misperception has mainly been studied in patients with insomnia [2,9]. However, recent studies have observed TST misperception in other sleep disorders such as obstructive sleep apnea (OSA) [10,11,12].

Studies on TST misperception in patients with OSA reported contradictory results, however, with some studies showing underestimation [10] and others, overestimation [13]. A reason for these inconsistent results may be related to the high heterogeneity of OSA [14,15]. For instance, many patients with OSA experience insomnia symptoms. The comorbid occurrence of OSA and insomnia or insomnia symptoms is often referred to as COMISA, with prevalences ranging from 30% to 50% in the OSA population [16,17]. Yet research on COMISA remains limited. It is plausible that the underestimation of TST in this population may be linked to the presence of insomnia symptoms. Some studies have shown that the presence of insomnia symptoms in patients with OSA is associated with the underestimation of TST [18,19]. Another study found no significant association between the presence of insomnia symptoms and TST misperception in this group [20]. This suggests that other factors beyond the presence of insomnia symptoms influence TST misperception in patients with OSA. For instance, a higher apnea–hypopnea index (AHI) was suggested to be linked to the overestimation of TST [18]. If this effect is not controlled for, the underestimating effect of insomnia symptoms on TST perception may be confounded by the overestimating effect of a high AHI. However, findings by Duarte and colleagues [10] suggested a different effect, with underestimation of TST increasing as AHI increases. Thus, further research is needed to clarify the factors associated with TST misperception in the OSA population.

Previous research on TST misperception in the OSA population predominantly relied on single-night polysomnography (PSG) studies. Since many sleep parameters show a high night-to-night variability [21,22] and many patients experience a first-night effect [21,23], a single measurement night is likely to give an incomplete picture of a patient’s sleep. Furthermore, during in-lab PSG assessment, the patient sleeps in the unnatural environment of a sleep laboratory. Even with ambulatory PSG, patients must sleep with many attached sensors, which can cause discomfort and alter their habitual sleeping conditions. These measurements may therefore not accurately reflect their regular sleep patterns and distort their subjective assessment of sleep.

Only a few multi-night studies on TST misperception in patients with insomnia symptoms have been conducted, all using actigraphy [24,25,26]. The studies suggested a high level of intra-individual variability in daily TST misperception, especially in people with sleep complaints, highlighting the importance of using multi-night measures. However, none of these studies included patients with OSA. Moreover, actigraphy itself has important drawbacks in this context, most notably its inability to measure wakefulness with high sensitivity. For instance, it is well known to misclassify motionless wake periods as sleep, and sleep periods with motion as wake, providing an erroneous picture of sleep efficiency, especially in the presence of sleep disorders [27,28].

Recent advances in sensor and analysis technologies have significantly improved wearable sleep monitoring using automatic sleep staging algorithms based on photoplethysmography (PPG) and accelerometer data obtained from wrist-worn devices [29,30]. The performance of this specific algorithm was evaluated in a large clinical population including patients with OSA and insomnia, reaching substantial agreement with sleep stages scored with gold standard PSG [29,30]. Accordingly, this method allows the acquisition of reliable TST estimates in an unobtrusive manner at home, over the course of multiple nights.

As discussed, previous single-night PSG studies have not always shown consistent results, possibly due to considerable night-to-night variability in sleep perception. The artificial environment and obtrusive sensors in PSG assessments may also limit the findings’ ecological validity. We hypothesized that further insights into this topic may be gained by leveraging recent advances in wearable technology to conduct a multi-night assessment of TST misperception in a more naturalistic environment.

Accordingly, we conducted a study in a cohort of recently diagnosed patients with OSA with and without insomnia symptoms, aiming to investigate factors associated with TST misperception with a multi-night approach using a wrist-worn PPG sensor which can reliably estimate sleep.

## 2. Results

### 2.1. Cohort and Baseline Clinical Characteristics

The present study made use of data collected in the Characterizing Sleep in COMISA (CHARISMA) study, a longitudinal observational study to characterize comorbid insomnia and sleep apnea (COMISA) [31]. Of the 241 OSA patients available in the CHARISMA dataset, 47 patients did not have a valid sleep diary available, and 53 patients had less than 2 nights of both subjective and objective recordings of sufficient quality available. Recordings were excluded because of incorrect or failed measurements, or incorrect data entry by the participants as detailed in the methods section. Consequently, 141 participants were included in the analysis. We compared the included and excluded patients on demographic characteristics (age, sex, BMI, AHI) and the proportion of patients experiencing insomnia symptoms and did not find any significant differences. The analyzed sample contained 49 women and 92 men. The median age was 50.00 years, with a minimum age of 24 years and a maximum age of 76 years. The baseline clinical characteristics of each group are summarized in Table 1. No significant differences in demographic characteristics were found between the two groups.

### 2.2. Multiple-Night Measurements per Group

**Figure 1:**
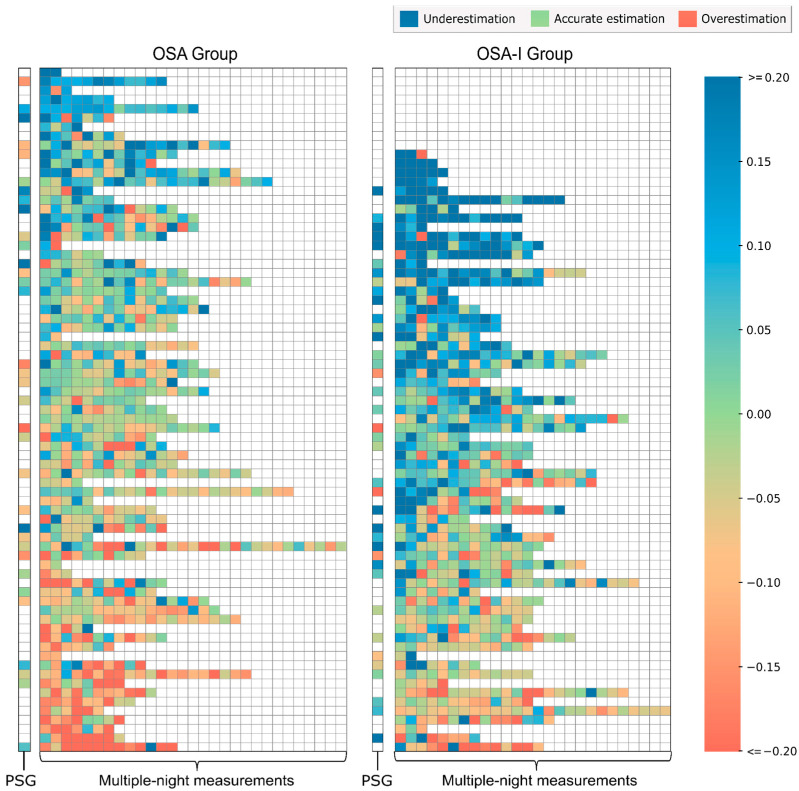
Daily MI values per group. Each row illustrates the misperception index (MI) values of a single participant. Rows were sorted on the respective median MI, sorted from top to bottom by decreasing values. Each square represents a single MI value. MI values are color-coded, with blue representing positive MI values (sleep time underestimation), green values representing values close to zero (accurate sleep time estimation), and red representing negative values (sleep time overestimation). For instance, an MI of 0.1 indicates an underestimation of TST by 10%. The first column for each group represents the MI value of the participant on the PSG night (if available). This is followed by the daily MI values recorded during the multi-night measurements. Daily MI values for each patient are arranged in chronological order, with each box representing the next available date if the directly following date is missing.

Although participants in both groups did not differ with regard to their objective TST, participants in the OSA-I group had lower self-reported TST values than participants in the OSA group (Table 2). Furthermore, a group comparison showed a significant difference in MI between the two groups. While participants in the OSA-I group tended to underestimate their TST (positive MI values), participants in the OSA group tended to overestimate it (negative MI values). For instance, patients in the OSA-I group showed a median TST underestimation of five percent. Daily MI values for each participant in both groups are depicted in Figure 1. A considerable amount of within- and between-participant variability in daily MI values was present. Consistent patterns of TST underestimation were more common in the OSA-I group while patterns of TST overestimation were more common in the OSA group. Moreover, participants in the OSA-I group rated their sleep quality lower than participants in the OSA group (Table 2). In Figure 1, we added the MI during the diagnostic PSG night, to show that TST misperception during the PSG night is not representative of the patterns observed in daily MI over the course of multiple nights. For example, patients with a comparable MI during the PSG night could show highly diverging patterns in daily MI during the subsequent nights.

### 2.3. Factors Associated with Daily MI Values

A linear mixed effects model revealed the following significant independent predictors of daily MI: presence of insomnia symptoms, AHI, and daily self-reported sleep quality. Specifically, the presence of insomnia symptoms was associated with higher daily MI values, and a higher AHI is associated with lower daily MI values (Table 3). Accordingly, participants who experience insomnia symptoms tended to underestimate their TST more than those who do not experience insomnia symptoms, and participants with a higher AHI tended to overestimate their TST more than participants with a lower AHI. Moreover, a lower daily self-reported sleep quality was associated with higher daily MI values. Thus, participants tend to underestimate their TST more on days on which they rate their sleep quality as worse. Visual inspection revealed that the distribution of residuals from the fitted model was approximately normal. The R-squared value [32] showed that the model explains approximately 38% of the variance in the dependent variable.

## 3. Discussion

Misperception of TST is associated with substantial adverse effects. However, research on the predictors of TST misperception in the OSA population has not been fully consistent, and existing studies have relied on single-night PSG. As was reported previously as well [24], the present study indicates that PSG may not yield a representative indicator of TST misperception at home on a night-to-night basis. To address this gap, we conducted the first study examining factors associated with TST misperception in OSA patients with and without insomnia symptoms using multi-night measurements in the home environment. To this end, we leveraged the recent development of a well-performing proxy measure of sleep based on wearable PPG/actigraphy. Our results highlight the contrasting effects of insomnia symptoms and AHI on TST misperception in patients with OSA.

Perceptions of TST diverged significantly between the OSA and the OSA-I group. While there was no significant difference between the groups with regard to the objective TST, a notable difference emerged in the self-reported TST. Consequently, a significant difference in median MI values was observed between the two groups, with participants with insomnia symptoms tending to underestimate their TST while participants without insomnia symptoms tended to overestimate it. Furthermore, the presence of insomnia symptoms emerged as a significant independent predictor of lower daily MI values, explaining a substantial amount of variance even after controlling for other factors such as AHI and self-reported sleep quality. Previous studies suggested that OSA patients with insomnia symptoms tend to underestimate their TST more than those without [18,19,33], but not all studies found insomnia symptoms to be a significant predictor of TST underestimation in the OSA population [20]. The results of the present study are in line with the hypothesis that insomnia symptoms are a predictor for TST underestimation, and we hypothesize that this was not consistently found in all previous work due to considerable night-to-night variability in patients’ sleep perception. Importantly, the results suggest that OSA patients with insomnia symptoms experience TST underestimation over the course of multiple nights in their regular sleeping environment. This underscores the significance of evaluating this issue in clinical practice.

In addition to insomnia symptoms, diagnostic AHI was shown to be a significant independent predictor of daily MI. Participants with a higher AHI tended to overestimate their sleep more. Previous research on the influence of AHI on TST misperception was inconsistent [10,18]. These findings seem to confirm the assumption that insomnia symptoms and severe OSA pull the perception of TST in opposite directions [18]. Accordingly, the present study offers a potential explanation for inconsistencies found previously: the perception of TST in patients with OSA may be influenced by other factors in addition to insomnia symptoms and confounded by factors such as AHI. In particular, the presence of a high AHI seems to convolute the relationship between insomnia symptoms and TST underestimation in the OSA population. Moreover, these findings demonstrate the existence of this pattern over the course of multiple nights in the home setting.

Apart from the constant effect of insomnia symptoms and AHI, the daily-varying factor of self-reported sleep quality emerged as a significant independent predictor of daily MI. Participants demonstrated a tendency to underestimate their TST more on days on which they rated their sleep quality lower. This suggests that daily variations in MI values are influenced by the participant’s experienced sleep quality. In essence, (longer-term) insomnia symptoms and the indication of low sleep quality for a particular night reflect similar sentiments, though they are not identical. One of the distinctions lies in the nuance that reporting insomnia symptoms encompasses a broader assessment, suggesting an overarching perception of consistently low sleep quality associated with difficulties falling or staying asleep over an extended period of time. Expressing a low sleep quality after a single night specifies a perceived low sleep quality on that particular date. While insomnia symptoms and low sleep quality are closely related, they both contribute to the variance explained by the linear mixed model, suggesting that both global and daily assessments of a patient’s sleep quality are independently valuable in predicting daily TST misperception. This underscores the value of integrating daily varying factors into the evaluation of TST misperception.

The age of the participants included in the study ranged from 24 to 76 years old, with the majority falling within a middle-age bracket but still representing a relatively broad age distribution. Furthermore, age was not found as a significant predictor of TST misperception in our analysis. This suggests that OSA patients’ perception of sleep is not substantially influenced by their age.

These findings have implications for clinical practice. For instance, the OSA-I group presenting insomnia symptoms at clinical intake showed a significant underestimation of TST. Comparably defined COMISA groups have been increasingly recognized in recent years [34]. Given that research in this population is limited so far, gaining insights into the sleep perception of COMISA patients is highly relevant. Our findings suggest that TST underestimation is more likely to occur in this population. While TST underestimation is associated with a lower quality of life [6] and greater functional disability [8], cognitive-behavioral therapy for insomnia (CBT-I) has been shown to improve the degree of TST underestimation in patients with insomnia symptoms [35]. The presence of comorbid OSA does not seem to impair the effectiveness of CBT-I [36]. Thus, it may be beneficial for COMISA patients to receive additional treatment beyond regular OSA management. Furthermore, COMISA is associated with lower continuous positive airway pressure (CPAP) adherence [34], yet it is unclear exactly by which aspect of insomnia this is caused. Therefore, it would be highly relevant to explore the relationship between TST misperception and CPAP adherence.

This work focused on pre-OSA treatment measurements. In future studies, it would be important to examine how TST misperception evolves during and after OSA treatment such as CPAP treatment, using long-term multi-night assessments. This could shed light on the impact of OSA treatment on TST misperception and the potential influence of misperception on treatment adherence. The impact of treatment on TST misperception may be especially relevant in patients experiencing both severe OSA and insomnia symptoms due to the opposing influence of these factors. As the AHI decreases during treatment, the influence of severe OSA may no longer mask the underestimation associated with the presence of insomnia symptoms. Therefore, it would be crucial to investigate how TST underestimation changes during the treatment of patients with severe OSA and insomnia symptoms. Additionally, the specific influence of comorbidities and medication use on the perception of sleep duration in OSA patients would be an important topic for future research.

In this manuscript, we chose to refer to the discrepancy between objective and subjective estimates of sleep duration as “TST misperception” since the misperception index [2] was used as the metric to describe this mismatch. However, it should be emphasized that it is not known if the discrepancy between objective and subjective measures results from the patient’s inaccurate perception of sleep or if the current standard sleep recording techniques do not capture ‘wake-like’ brain activity, which causes the feeling of being awake [37].

Because of its retrospective and cross-sectional nature, it is not possible to draw conclusions on temporal or causal relationships. While demographic variables such as age and gender were not found to be related to sleep perception in this study, we cannot exclude center-specific biases. Accordingly, to ensure the generalizability of the present findings to patients in different sleep centers, countries, and specific age groups, the analyses should be replicated in different cohorts. Furthermore, the conclusions of this study need to also be verified in future studies including patients with a formal COMISA diagnosis.

The present study required a simultaneous measurement of both subjective and objective sleep. Due to the largely unsupervised home measurement setup, and despite the attempts to minimize its impacts such as clear instructions for use, and regular calls from the investigators, it was unavoidable that a relatively large portion of subjects had to be excluded from the analysis due to incomplete data. However, a comparison of available demographic and clinical characteristics revealed that this excluded portion did not significantly differ from the included cohort.

Furthermore, we believe that the validity of this home measurement approach, in contrast with, e.g., a well-controlled but artificial setting such as multi-night monitoring in the lab, outweighs such disadvantages. We recognize that the measurement modality used in the present study is less precise than PSG. However, it offers much greater accuracy than actigraphy and enables multi-night assessments in a naturalistic environment, which PSG cannot provide. The algorithm’s previous validation against PSG within this cohort further supports its accuracy, as evidenced by its ability to identify significant predictors of sleep perception in OSA.

In conclusion, differences in TST misperception in OSA patients with and without insomnia symptoms can be found across multiple nights using validated proxy sleep measurements at home. Underestimation of TST is more common in OSA patients with insomnia symptoms than in those without. Furthermore, the presence of insomnia symptoms is predictive of higher MI, corresponding to increased TST underestimation, while higher diagnostic AHI values predict lower MI or increased TST overestimation. In addition, this study demonstrates the importance of incorporating daily varying factors such as self-reported sleep quality into the analysis since it is an independent predictor of MI, even after accounting for other fixed parameters.

## 4. Materials and Methods

Data were collected in the Characterizing Sleep in COMISA (CHARISMA) study, a longitudinal observational study to characterize comorbid insomnia and sleep apnea (COMISA) in a cohort of patients referred due to the suspicion of OSA, scheduled for PSG as part of the standard clinical routine who were then monitored over the course of two weeks at home [31]. Recruitment took place at the Amphia Center for Sleep Medicine, Breda, The Netherlands, a second-line sleep center. Participants were excluded from the CHARISMA study if they were younger than 18 years old, had known autonomic dysfunction or persistent heart rhythm disorders, used alpha-blocking medication, or were pregnant. The study (W20.090) was approved by the medical ethical committee of the Maxima Medical Center (Veldhoven, The Netherlands, N16.074). All participants provided written informed consent.

We selected two groups of patients from the CHARISMA dataset. The ‘OSA group’ consisted of patients with a formal diagnosis of OSA, who did not report any complaints regarding sleep onset and/or sleep maintenance in the clinical interview. The ‘OSA-I group’ included the patients who received a formal diagnosis of OSA and reported problems regarding sleep onset and/or sleep maintenance in the clinical interview. All assessments regarding insomnia symptoms were performed by a clinical somnologist during clinical intake.

### 4.1. Baseline Measures

The following data were obtained from the clinical record: age (in years), sex, body mass index (BMI), AHI, and OSA diagnosis (diagnosed by a somnologist according to International Classification of Sleep Disorders criteria [38]). AHI and OSA diagnoses were based on ambulatory diagnostic PSG. Respiratory events were scored by a certified sleep technician following the American Academy of Sleep Medicine guidelines [39]. Hypopnea was scored when there was an oxygen desaturation of 3% or higher and/or when the event was associated with an arousal.

Objective TST was also scored from the ambulatory PSG night. Reported TST during the PSG night was obtained through the digital sleep diary as explained in the daily measures section.

### 4.2. Daily Measures

Participants were asked to complete two daily measures for a period of two weeks, starting the day after the diagnostic PSG: (1) a measurement during the night, using a wrist-worn PPG and accelerometer sensor, to obtain objective TST, and (2) a digital sleep diary (filled-in in the morning) to obtain self-reported TST. Participants were free to continue with the daily measurements after the two-week period had passed. All measurements were completed before participants received their diagnosis and, when applicable, before starting with treatment.

#### 4.2.1. Objective Sleep Measures

Nightly measurements were performed with a CE-marked wrist-worn logging device (Philips Research, Eindhoven, The Netherlands). The device contains a PPG sensor including two green LEDs, a photodiode (sampling frequency: 32 Hz), and a three-axial accelerometer (sampling frequency: 32 Hz). Participants wore the device on their wrists with the sensor oriented towards the skin on the dorsal side of the hand. Participants were asked to put on the device every evening before going to bed. They did not have access to the data acquired through the wrist-worn sensor and the data were not used for any part of the clinical assessment, or diagnostic procedure.

Objective TST was derived using a 4-class sleep staging algorithm that used as input instantaneous heart rate calculated from interbeat intervals detected from the PPG signal and body movements measured from the accelerometer [30]. The performance of this algorithm for automatic sleep stage classification was previously validated in a large clinical cohort, showing reliable performance even in patients with different sleep pathologies [30]. In comparison with manually scored sleep stages based on PSG, the algorithm achieved a Cohen’s κ coefficient of agreement of 0.64 [30]. The performance of this algorithm was also previously verified in the single-night PSG recording of the CHARISMA dataset, achieving a Cohen’s κ coefficient of agreement of 0.65, confirming its accuracy for this task [31].

#### 4.2.2. Self-Reported Sleep Parameters

Self-reported parameters were collected using a digital sleep diary which was developed by the Kempenhaeghe Center for Sleep Medicine, Heeze, The Netherlands. Participants could access the sleep diary through an application installed on a smartphone that was provided to each participant. Participants were asked to fill in the sleep diary every morning upon waking up, for a period of two weeks. Participants were able to enter data for the immediately preceding night in the sleep diary, but they were not allowed to enter data for nights that occurred further in the past. Participants could review all data they had previously entered in the sleep diary, but they were only allowed to change the entry for the immediately preceding night. Entries made further in the past could not be changed retrospectively.

Using the application’s graphical interface, participants noted each morning: (1) the time at which they wanted to go to sleep, (2) the time at which they wanted to get up, and (3) at what moments during the night they were asleep, awake in bed, awake out of bed, all with a resolution of 5 min. Finally, they were asked to rate their experienced quality of sleep on a continuous visual slider line, from ‘very bad’ to ‘excellent’, numerically quantified on a scale from 0 to 1.

Self-reported TST was calculated as the total number of minutes that the participant indicated being asleep between the time the participant wished to fall asleep and the time at which the participant wished to get up.

#### 4.2.3. Exclusion Criteria

To ensure sufficient data quality, certain recordings were excluded from the analysis. From the objective sleep measures, recordings with an interbeat interval (IBI) coverage of less than 75% and/or a total recording time of less than four hours were excluded from the analysis. IBI coverage is a direct reflection of the ability to detect heartbeats from the PPG signal and is impacted mainly by two factors. The first occurs when the optical sensor has poor coupling with the participant’s skin and usually results in a PPG signal with poor quality, usually with excessively low amplitude or a poor signal-to-noise ratio. The second occurs due to movement artifacts which distort the waveform. In periods when either of these occurs, heartbeats are simply not detected, resulting in (long) time intervals without IBIs. By comparing the total PPG recording duration with the sum of all detected IBIs, we can calculate the IBI coverage as the percentage of recording time with detected heartbeats. Using a minimum of 75% IBI coverage as a criterion, we exclude recordings where the low signal quality is most likely related to sensor disconnection or poor participant coupling—a problem typically present throughout a large portion of the recording. At the same time, this will allow us to retain recordings where the lower signal quality is more probably due to movement artifacts, unlikely to last more than a total of 25% of the night, and which are relevant in the context of the present study as they might be reflective of periods of wakefulness, which we expect to encounter in this population.

From the sleep diary recordings, sessions for which participants reported a TST of 0 min but indicated a sleep quality higher than 0.1, and sessions in which participants reported less than 60 min of TST and a sleep quality higher than 0.3, were excluded since these combinations of scores are highly unlikely and suggest an error when filling in the sleep diary.

To effectively capture night-to-night variability in TST misperception in our dataset, we chose participants who had a minimum number of two recording nights including simultaneous objective sleep measurements and sleep diary recordings.

### 4.3. Misperception Index (MI) Calculation

TST misperception was quantified using the misperception index (MI) [2]. The MI is computed according to the following formula:

MI = (objective TST − self-reported TST)/objective TST

An MI value of 0 indicates a perfect estimation of TST, positive values indicate an underestimation of TST, and negative values an overestimation of TST. For instance, an MI of 0.1 indicates an underestimation of TST by 10%. This measure has been shown to reliably describe the main features of TST misperception in healthy and sleep-disordered participants. In particular, the MI is also sensitive to the identification of TST underestimation on nights on which participants reported not having slept even when their objective TST was low.

### 4.4. Statistical Analysis

Baseline clinical characteristics and multiple-night measurements were reported for both groups. A Shapiro–Wilk test was used to assess the normality of all variables. Since the majority of variables were not normally distributed, we decided to report all variables as median, with first and third quartiles (Q1 and Q3, respectively). For parameters with multiple measurements per participant (objective TST, self-reported TST, MI, and self-reported sleep quality), median values were first calculated per participant to correct for the different number of observations available per participant. Accordingly, group-based sample statistics for these parameters are then reported as medians of medians. Between-group comparisons for continuous variables were performed using the non-parametric Mann–Whitney U test, and categorical variables were analyzed using the chi-square test.

Factors associated with daily MI were evaluated using a linear mixed-effects model. The benefit of using such models to analyze multi-night data is that they can cope with the fact that repeated measures within participants are not independent. Accordingly, the linear mixed model allows us to consider all available data for each participant while being robust to randomly missing data and to a different number of measurements between participants. In the mixed model, the presence of insomnia symptoms was included as a regressor. Since demographic and clinical characteristics are plausibly associated with TST misperception, we further included age (in years) [18], sex [40], and AHI [18], as covariates in the model. Additionally, we included self-reported sleep quality as a daily covariate. Participant identifiers were used as a random effect in the model, using random intercepts.

The linear mixed effects model was fitted and evaluated in R (version 4.3.1) [41] using the package lme4 (version 1.1.34) [42]. All remaining statistical analyses were carried out using Python (version 3.7) [43].

Since several variables were compared across the same groups, significance was established using the Benjamini–Hochberg procedure [44], with a maximum acceptable false discovery rate of 10%. Accordingly, the comparison k with the largest *p*-value still below the critical P(k) was considered significant. All comparisons with a rank below k were also considered significant.

## Figures and Tables

**Table 1:** Cohort description.

	OSA (*n* = 75)	OSA-I (*n* = 66)	*p*
Age, years	51.00 (41.50–56.50)	49.50 (45.00–58.00)	0.602
Male, % male	53 (70.67)	39 (59.09)	0.207
BMI, kg/m^2^	28.10 (26.09–32.48)	28.78 (25.73–33.14)	0.793
AHI, events/h	20.40 (11.05–32.95)	17.20 (11.43–28.68)	0.502

Values presented as median (interquartile range), or number (%). AHI = apnea–hypopnea index, BMI = body mass index, OSA = obstructive sleep apnea.

**Table 2:** Multiple-night measurements per group.

	OSA (*n* = 75)	OSA-I (*n* = 66)	*p*
Number of recording nights	12.00 (7.00–15.00)	12.50 (8.00–14.75)	0.558
Objective TST, min	416.50 (388.25–436.25)	410.25 (377.94–445.13)	0.861
Reported TST, min	415.00 (390.00–456.25)	386.25 (360.00–420.00)	<0.001 *
Misperception index (MI)	−0.02 (−0.06–0.02)	0.05 (−0.02–0.13)	<0.001 *
Reported sleep quality	0.63 (0.56–0.77)	0.52 (0.46–0.61)	<0.001 *

Values presented as median with first and third quartile (Q1–Q3). MI = Misperception Index, TST = total sleep time, OSA = obstructive sleep apnea. * Indicates significant difference after Benjamini–Hochberg control for false discovery rate.

**Table 3:** Univariate linear mixed model analysis predicting daily MI values.

Effect Type	Variables	C	SE	*p*
Constant effects	Intercept	7.687 × 10^−2^	5.494 × 10^−2^	0.164
	Age	1.315 × 10^−3^	9.395 × 10^−4^	0.164
	Sex	1.101 × 10^−2^	2.058 × 10^−2^	0.594
	AHI	−1.286 × 10^−3^	4.589 × 10^−4^	0.006 *
	Insomnia symptoms	6.960 × 10^−2^	1.978 × 10^−2^	<0.001 *
Variable effects	Reported sleep quality	−2.287 × 10^−1^	2.513 × 10^−2^	<0.001 *

Constant effects = one measurement per participant during the clinical intake, Variable effects = daily measurements for each participant, C = coefficient of the predictor, SE = standard error, *p* = *p*-value, AHI = apnea–hypopnea index. * Indicates significant difference after Benjamini–Hochberg control for false discovery rate.

## Data Availability

The data that support the findings of this study are not publicly available due to privacy and legal restrictions. Requests to access the data should be directed to the corresponding author.
